# Supplementary material for: Convergent evolution of SARS-CoV-2 Omicron subvariants leading to the emergence of BQ.1.1 variant
Source: Nat Commun. 2023 May 11;14:2671. doi: 10.1038/s41467-023-38188-z (PMC10175283; doi:10.1038/s41467-023-38188-z)
Supplement: Supplementary file 1 — Supplementary Information [file 41467_2023_38188_MOESM1_ESM.pdf]

Supplementary Table 1. Information on the number of estimated substitution events in each Omicron lineage

Lineage	Site	Substitution events #	Total sequences	Events per 1M sequences
BA.1	F486	0	1,273,375	0.0
BA.1	K444	3	1,273,375	2.4
BA.1	L452	4	1,273,375	3.1
BA.1	N460	3	1,273,375	2.4
BA.1	R346	16	1,273,375	12.6
BA.2	F486	6	1,603,935	3.7
BA.2	K444	13	1,603,935	8.1
BA.2	L452	27	1,603,935	16.8
BA.2	R346	9	1,603,935	5.6
BA.4	K444	5	115,119	43.4
BA.4	N460	1	115,119	8.7
BA.4	R346	11	115,119	95.6
BA.5	K444	65	837,977	77.6
BA.5	N460	12	837,977	14.3
BA.5	R346	49	837,977	58.5
BA.2 (excluding BA.2.75)	F486	1	1,597,209	0.6
BA.2 (excluding BA.2.75)	K444	11	1,597,209	6.9
BA.2 (excluding BA.2.75)	L452	24	1,597,209	15.0
BA.2 (excluding BA.2.75)	N460	3	1,597,209	1.9
BA.2 (excluding BA.2.75)	R346	11	1,597,209	6.9
BA.2.75	F486	5	6,726	743.4
BA.2.75	K444	2	6,726	297.4
BA.2.75	L452	3	6,726	446.0
BA.2.75	R346	6	6,726	892.1

Supplementary Table 2. Crystallographic data collection and refinement statistics

	BQ.1.1 RBD-ACE2 Complex
**Data collection**	
Space group	$P3_212$
Cell dimensions	
a, *b*, *c* (Å)	120.05, 120.05, 167.42
Resolution (Å)	49.17-2.78 (2.95-2.78) *
R_{meas} (%)	26.1 (313.7)
$CC_{1/2}$ (%)	99.8 (41.8)
$<I / \sigma(I)>$	14.4 (1.03)
Completeness (%)	99.7 (98.6)
Redundancy	20.7 (17.9)
**Refinement**	
No. reflections	34681
R_{work} / R_{free} (%)	20.0/24.5
No. atoms	
Protein	6440
Ligand	243
Water	30
B-factors (Å^2)	
Protein	81.98
Ligand/ion	119.4
Water	53.3
R.m.s. deviations	
Bond lengths (Å)	0.002
Bond angles (°)	0.547
Ramchandran Statistics (%)	
Favored	96.84
Outliers	0.13

*Values in parentheses are for highest-resolution shell.

Supplementary Table 3. Human sera used in this study

Donor ID	Sex	Age	vaccine (1st vaccination)	Date of 1st vaccination (YYYY-MM-DD)	vaccine (2nd vaccination)	Date of 2nd vaccination (YYYY-MM-DD)	vaccine (3rd vaccination)	Date of 3rd vaccination (YYYY-MM-DD)	vaccine (4th vaccination)	Date of 4th vaccination (YYYY-MM-DD)	vaccine (5th vaccination)	Date of 5th vaccination (YYYY-MM-DD)	Date of sampling (YYYY-MM-DD)	Prior infection?
4	Female	43	BNT162b2	2021-05-14	BNT162b2	2021-06-04	BNT162b2	2022-01-28	BNT162b2	2022-08-01			2022-08-22	No
8	Female	56	BNT162b2	2021-05-14	BNT162b2	2021-06-04	BNT162b2	2022-01-28	BNT162b2	2022-08-05			2022-08-26	No
14	Female	43	BNT162b2	2021-05-14	BNT162b2	2021-06-04	BNT162b2	2022-01-28	BNT162b2	2022-08-05			2022-08-26	No
28	Female	42	BNT162b2	2021-04-06	BNT162b2	2021-04-28	BNT162b2	2022-01-22	BNT162b2	2022-07-30			2022-08-23	No
61	Female	48	BNT162b2	2021-05-14	BNT162b2	2021-06-04	BNT162b2	2022-01-29	BNT162b2	2022-08-05			2022-09-06	No
62	Male	46	BNT162b2	2021-05-14	BNT162b2	2021-06-10	BNT162b2	2022-01-28	BNT162b2	2022-08-05			2022-08-26	No
63	Female	49	BNT162b2	2021-05-12	BNT162b2	2021-05-31	BNT162b2	2022-02-05	BNT162b2	2022-08-02			2022-08-25	No
64	Female	42	BNT162b2	2021-05-10	BNT162b2	2021-05-31	BNT162b2	2022-02-03	BNT162b2	2022-08-01			2022-08-25	No
67	Male	38	BNT162b2	2021-05-11	BNT162b2	2021-06-01	BNT162b2	2022-02-01	BNT162b2	2022-08-03			2022-08-24	No
69	Male	42	BNT162b2	2021-06-11	BNT162b2	2021-07-17	BNT162b2	2022-01-28	BNT162b2	2022-08-10			2022-09-06	No
71	Male	34	BNT162b2	2021-03-18	BNT162b2	2021-04-08	BNT162b2	2021-12-28	BNT162b2	2022-08-09			2022-09-06	No
74	Male	28	BNT162b2	2021-03-26	BNT162b2	2021-04-16	BNT162b2	2022-01-28	BNT162b2	2022-08-09			2022-09-07	No
90	Male	45	BNT162b2	2021-05-11	BNT162b2	2021-06-02	BNT162b2	2022-01-25	BNT162b2	2022-08-02			2022-08-24	No
92	Female	30	BNT162b2	2021-05-20	BNT162b2	2021-06-10	BNT162b2	2022-01-26	BNT162b2	2022-08-04			2022-08-26	No
94	Male	36	BNT162b2	2021-05-11	BNT162b2	2021-06-01	BNT162b2	2022-02-02	BNT162b2	2022-08-05			2022-08-26	No
97	Female	43	BNT162b2	2021-05-12	BNT162b2	2021-06-04	BNT162b2	2022-01-26	BNT162b2	2022-07-21			2022-08-19	No
103	Male	35	BNT162b2	2021-03-17	BNT162b2	2021-04-07	BNT162b2	2022-01-25	BNT162b2	2022-08-02			2022-08-26	No
113	Female	32	BNT162b2	2021-05-13	BNT162b2	2021-06-04	BNT162b2	2022-01-28	BNT162b2	2022-08-02			2022-08-23	No
122	Female	45	BNT162b2	2021-05-20	BNT162b2	2021-06-11	BNT162b2	2022-02-16	BNT162b2	2022-08-01			2022-08-22	No
115117	Male	65	BNT162b2	2021-08-01	BNT162b2	2021-08-22	BNT162b2	2022-03-25	BNT162b2 (BA.1)	2022-10-01			2022-10-22	No
210718	Female	55	NA	NA	NA	NA	NA	2022-04-10	BNT162b2 (BA.1)	2022-10-02			2022-10-23	No
331843	Male	58	NA	2021-08-22	NA	2021-10-03	BNT162b2	2022-04-23	BNT162b2 (BA.1)	2022-10-03			2022-10-23	No
2362037	Male	57	NA	NA	NA	2021-08-29	BNT162b2	2022-04-07	BNT162b2 (BA.1)	2022-09-29			2022-10-21	No
108127	Female	57	BNT162b2	2021-08-07	BNT162b2	2021-09-08	BNT162b2	2022-04-07	BNT162b2 (BA.1)	2022-09-30			2022-10-21	No
190497	Female	52	BNT162b2	2021-08-30	BNT162b2	2021-09-21	mRNA-1273	2022-03-28	BNT162b2 (BA.1)	2022-10-01			2022-10-23	No
1437	Female	73	BNT162b2	2021-07-31	BNT162b2	2021-09-08	BNT162b2	2022-04-24	BNT162b2 (BA.1)	2022-09-28			2022-10-22	No
247029	Female	50	NA	2021-09-21	NA	2021-10-20	BNT162b2	2022-04-28	BNT162b2 (BA.1)	2022-09-28			2022-10-21	No
2382760	Female	30	mRNA-1273	2021-07-08	BNT162b2	2021-10-10	BNT162b2	2022-04-21	BNT162b2 (BA.1)	2022-09-28			2022-10-17	No
129061	Male	42	NA	NA	NA	NA	BNT162b2	2022-04-14	BNT162b2 (BA.1)	2022-10-01			2022-10-22	No
318009	Female	58	BNT162b2	2021-08-06	BNT162b2	2021-08-30	BNT162b2	2022-03-18	BNT162b2 (BA.1)	2022-09-29			2022-10-26	No
218082	Female	45	BNT162b2	2021-09-24	BNT162b2	2021-10-15	BNT162b2	2022-04-16	BNT162b2 (BA.1)	2022-09-30			2022-10-29	No
7371	Female	45	NA	NA	NA	NA	mRNA-1273	2022-04-23	BNT162b2 (BA.1)	2022-10-08			2022-10-30	No
145410	Male	71	BNT162b2	2021-07-23	BNT162b2	2021-08-23	BNT162b2	2022-03-18	BNT162b2 (BA.1)	2022-10-08			2022-10-29	No
2362083	Female	57	BNT162b2	2021-09-26	BNT162b2	2021-10-17	BNT162b2	2022-04-17	BNT162b2 (BA.1)	2022-10-08			2022-10-29	No
2420192	Female	36	BNT162b2	2021-03-08	BNT162b2	2021-03-29	BNT162b2	2021-12-10	BNT162b2 (BA.1)	2022-10-08			2022-10-29	No
149621	Female	80	BNT162b2	2021-06-29	BNT162b2	2021-07-28	BNT162b2	2022-03-04	BNT162b2 (BA.1)	2022-10-07			2022-10-28	No
309473	Female	57	NA	NA	NA	2021-09-17	NA	NA	BNT162b2 (BA.1)	2022-10-07			2022-11-01	No
2385248	Male	56	NA	NA	NA	NA	BNT162b2	2022-05-18	BNT162b2 (BA.1)	2022-10-06			2022-10-30	Yes (2021-08-14)
2362137	Male	63	NA	NA	NA	NA	mRNA-1273	2022-03-04	BNT162b2 (BA.1)	2022-10-06			2022-10-29	No
15548	Male	55	NA	NA	NA	NA	mRNA-1273	2022-04-13	BNT162b2 (BA.1)	2022-10-06			2022-11-19	No
2372779	Female	37	BNT162b2	2021-04-27	BNT162b2	2021-05-18	BNT162b2	2022-01-14	BNT162b2 (BA.1)	2022-09-30			2022-11-01	NO
KS221117	Male	40	BNT162b2	2021-06-17	BNT162b2	2022-07-07	mRNA-1273	2022-03-28	BNT162b2 (BA.5)	2022-10-27			2022-11-17	No
216850	Female	59	BNT162b2	2021-09-04	BNT162b2	2021-09-25	BNT162b2	2022-04-07	BNT162b2 (BA.5)	2022-10-27			2022-11-21	No
226147	Male	49	BNT162b2	2021-09-21	BNT162b2	2021-10-20	BNT162b2	2022-05-21	BNT162b2 (BA.5)	2022-10-27			2022-11-21	No
2422938	Female	27	BNT162b2	2021-09-10	BNT162b2	2021-11-02	BNT162b2	2022-05-	BNT162b2 (BA.5)	2022-10-31			2022-11-22	No
2422916	Female	54	BNT162b2	2021-09-28	BNT162b2	2021-10-19	BNT162b2 (BA.5)	2022-10-31					2022-11-22	No
2422887	Male	32	BNT162b2	2021-08-23	mRNA-1273	2021-09-21	mRNA-1273	2022-03-28	BNT162b2 (BA.5)	2022-10-31			2022-11-22	Yes (2021-08-)
2404750	Female	51	BNT162b2	2021-07-23 or 2021-07-24	BNT162b2	2021-08-12	BNT162b2	2022-03-16	BNT162b2 (BA.5)	2022-10-31			2022-11-22	Yes (2022-07-08)
2363002	Male	52	BNT162b2	2021-08-27	BNT162b2	2021-09-17	BNT162b2	2022-04-09	BNT162b2 (BA.5)	2022-10-29			2022-11-23	No
1053	Male	86	BNT162b2	2021-05-17	NA	2021-06-07	NA	NA	NA	NA	BNT162b2 (BA.5)	2022-11-01	2022-11-24	No
2420132	Female	50	BNT162b2	2021-08-27	BNT162b2	2021-09-17	BNT162b2	2022-03-24	BNT162b2 (BA.5)	2022-10-28			2022-11-23	No
2423214	Male	77	BNT162b2	2021-03-	NA	2021-03-	NA	2022-03-29	NA	2022-07-05	BNT162b2 (BA.5)	2022-11-01	2022-11-24	No
2423218	Female	82	BNT162b2	2021-03-	NA	2021-03-	NA	2022-03-29	NA	2022-07-05	BNT162b2 (BA.5)	2022-11-01	2022-11-24	No
233746	Male	52	BNT162b2	2021-07-	NA	2021-07-	NA	2022-03-	BNT162b2 (BA.5)	2022-10-31			2022-11-24	No
NM221207	Female	54	BNT162b2	2021-06-26	BNT162b2	2021-07-17	BNT162b2	2022-02-05	BNT162b2 (BA.5)	2022-11-16			2022-12-07	No
2366112	Female	42	NA	2021-10-01	NA	2021-10-	NA	2022-05-01	BNT162b2 (BA.5)	2022-11-01			2022-11-25	No
14137	Female	41	BNT162b2	2021-09-10	BNT162b2	2021-10-01	BNT162b2	2022-04-16	BNT162b2 (BA.5)	2022-10-29			2022-11-27	No
2418539	Female	72	NA	NA	NA	NA	BNT162b2	2021-07-22	BNT162b2 (BA.5)	2022-11-06			2022-11-27	No
2420249	Male	47	BNT162b2	2021-09-05	BNT162b2	2021-09-26	BNT162b2	2022-04-10	BNT162b2 (BA.5)	2022-10-30			2022-11-27	No
2423718	Female	49	BNT162b2	2021-09-	BNT162b2	2022-01-	BNT162b2	2022-04-	BNT162b2 (BA.5)	2022-11-03			2022-11-27	No
319561	Male	18	BNT162b2	2021-09-21	BNT162b2	2021-10-12	BNT162b2	2022-04-15	BNT162b2 (BA.5)	2022-11-01			2022-11-27	No
2372043	Male	43	NA	NA	NA	NA	BNT162b2	2022-05-26	BNT162b2 (BA.5)	2022-11-01			2022-11-28	No

SARS-CoV-2 infected	Donor ID	Sex	Age	Date of test (YYYY/MM/DD)	Date of sampling (YYYY/MM/DD)	Prior infection?	Prior vaccination?	Vaccine	Date of 1st vaccination (YYYY/MM/DD)	Date of 2nd vaccination (YYYY/MM/DD)	Date of 3rd vaccination (YYYY/MM/DD)	Date of 4rd vaccination (YYYY/MM/DD)
BA.2	P378	Male	43	2022/03/28	2022/04/10	No	Yes	BNT162b2	2021/10/10	2021/10/31		
BA.2	P398	Male	48	2022/04/13	2022/04/30	No	Yes	BNT162b2	2021/09/18	2021/10/09	2022/04/09	
BA.2	P407	Male	29	2022/05/01	2022/05/12	No	Yes	mRNA-1273	2021/09/13	2021/10/11		
BA.2	P401	Male	35	2022/04/22	2022/05/05	No	Yes	BNT162b2	2021/09/09	2021/09/30		
BA.2	P412	Female	82	2022/05/04	2022/05/26	No	Yes	BNT162b2	2021/06/11	2021/07/09		
BA.2	6449	Male	43	2022/04/03	2022/04/23	No	Yes	BNT162b2	2021/08/13	2021/09/11		
BA.2	6355	Male	50	2022/04/02	2022/04/20	No	Yes	NA	2021/04/28	2021/05/19	2022/01/19	
BA.2	6547	Male	54	2022/04/06	2022/04/22	No	Yes	BNT162b2	2021/08/25	2021/09/15		
BA.2	7951	Female	71	2022/04/25	2022/05/12	No	Yes	Mix	2021/06/20 (BNT162b2)	2021/07/16 (BNT162b2)	2022/02/16 (mRNA-1273）	
BA.2	8645	Female	41	2022/05/07	2022/05/20	No	Yes	BNT162b2	2021/05/23	2021/06/13	2022/01/20	
BA.2	8682	Female	25	2022/05/08	2022/05/24	No	Yes	BNT162b2	2021/09/03	2021/09/27		
BA.2	5949	Male	24	2022/03/22	2022/05/22	No	Yes	mRNA-1273	2021/08/05	2021/09/02		
BA.2	8463	Male	84	2022/05/05	2022/05/23	No	Yes	Mix	2021/05/27 (BNT162b2)	2021/06/21 (BNT162b2)	2022/02/22(mRNA-1273）	
BA.2	8796	Female	34	2022/05/10	2022/06/05	No	Yes	BNT162b2	2021/09/26	2021/10/17		
BA.5	P427	Female	49	2022/07/06	2022/07/25	No	Yes	NA	2021/07/30	2021/08/25	2022/03/18	
BA.5	P440	Male	25	2022/07/24	2022/08/07	No	Yes	BNT162b2	2021/11/24	2021/12/15		
BA.5	P439	Female	73	2022/07/23	2022/08/08	No	Yes	BNT162b2	2021/06/19	2021/07/20	2022/02/04 (mRNA-1273)	
BA.5	P451	Female	55	2022/07/29	2022/8/12	No	Yes	BNT162b2	2021/04/26	2021/05/20	2022/01/18	
BA.5	P456	Male	44	2022/08/04	2022/08/14	No	Yes	BNT162b2	2021/08/11	2021/09/01	2022/03/13 (mRNA-1273)	
BA.5	P455	Male	29	2022/08/03	2022/08/17	No	Yes	mRNA-1273	2021/07/26	2021/08/27	2022/04/18	
BA.5	P464	Male	63	2022/08/08	2022/08/19	No	Yes	BNT162b2	2021/08/08	2021/08/29	2022/04/07 (mRNA-1273)	
BA.5	9341	Male	56	2022/06/12	2022/06/30	No	Yes	BNT162b2	2021/08/10	2021/08/31	2022/03/18	
BA.5	9584	Male	55	2022/07/08	2022/07/25	No	Yes	BNT162b2	2021/07/14	2021/08/05	2022/03/22	
BA.5	11318	Female	51	2022/07/24	2022/08/05	No	Yes	BNT162b2	2021/09/01	2021/09/22	2022/05/19 (mRNA-1273)	
BA.5	23S-08	Male	25	2022/07/23	2022/08/08	No	Yes	BNT162b2	2021/04/27	2021/05/18	2022/01/11	
BA.5	11597	Female	41	2022/07/26	2022/08/08	No	Yes	BNT162b2	2021/04/30	2021/05/21	2022/01/11	
BA.5	10978	Female	46	2022/07/22	2022/08/11	No	Yes	BNT162b2	2021/08/27	2021/09/17	2022/05/15	
BA.5	10826	Male	63	2022/07/21	2022/08/11	No	Yes	BNT162b2	2021/07/27	2021/08/17	2022/03/04	2022/8/9 (mRNA-1273)
BA.5	11079	Female	65	2022/07/23	2022/08/11	No	Yes	mRNA-1273	2021/07/08	2021/08/05	2022/03/17	
BA.5	14847	Female	70	2022/08/13	2022/08/25	No	Yes	BNT162b2	2021/07/13	2021/08/20	2022/03/08 (mRNA-1273)	
BA.5	13180	Female	63	2022/08/04	2022/08/25	No	Yes	BNT162b2	2021/07/16	2021/08/06	2022/03/08 (mRNA-1273)	
BA.5	12912	Male	64	2022/08/02	2022/08/25	No	Yes	mRNA-1273	2021/09/02	2021/09/30	2021/04/01	
BA.5	14956	Female	33	2022/08/13	2022/08/28	No	Yes	BNT162b2	2021/09/06	2021/10/07		
BA.5	15707	Female	52	2022/08/16	2022/08/29	No	Yes	BNT162b2	2021/08/07	2021/08/28	2022/04/03	

NA, not applicable.

Supplementary Table 4. Primers used in this study

Primer name	Primer sequence (5'-to-3')	Purpose
Omicron universal Fw	cactatagggcgaattgggtaccatgtttgtgttcctggt	Preparation of S expression plasmid
BA.2 WT Rv	agctccaccgcggtggcggccgctcaggtgtagtgcagtttca	Preparation of S expression plasmid
pC-S_BA2-R346T_R	atagacagaggcaaaGGTggtggcattgaacac	Preparation of S expression plasmid
pC-S_BA2-R346T_F	gtgttcaatgccaccACCtttgcctctgtctat	Preparation of S expression plasmid
pC-S_BA2-K444T_R	gtagttgcctcccacGGTgctgtccagcttgtt	Preparation of S expression plasmid
pC-S_BA2-K444T_F	aacaagctggacagcACCgtgggaggcaactac	Preparation of S expression plasmid
pC_S_BA2_N460K_R	aaatggtttcagCTTgctcttcctgaa	Preparation of S expression plasmid
pC_S_BA2_N460K_F	ttcaggaagagcAAGctgaaaccattt	Preparation of S expression plasmid

Supplementary Table 5. Summary of unexpected amino acid mutations detected in the working virus stocks

Original clinical isolate	Reference	Position in Reference (nt)	REF	ALT	Quality score	Depth in REF	Depth in ALT	Mutation type	ORF	Amino acid substitution
BA.2 (EPI_ISL_9595859)	No additional amino acid substitutions were detected.									
BA.5 (EPI_ISL_12812500)	NC_045512.2	2,388	C	T	228	6	209	missense_variant	NSP2	T528I
BA.5 (EPI_ISL_12812500)	NC_045512.2	11,750	C	T	222	151	96	missense_variant	NSP6	L260F
BQ.1.1 (EPI_ISL_15579783)	NC_045512.2	26,283	AGTT	A	222	137	93	conservative_inframe_deletion	E	V14del
Delta (EPI_ISL_2378732)	NC_045512.2	17,440	C	T	228	12	122	missense_variant	NSP13	P402S
Delta (EPI_ISL_2378732)	NC_045512.2	19,010	A	G	228	15	137	missense_variant	NSP14	D324G
Delta (EPI_ISL_2378732)	NC_045512.2	27,832	T	A	148	182	60	missense_variant	ORF7b	I26N

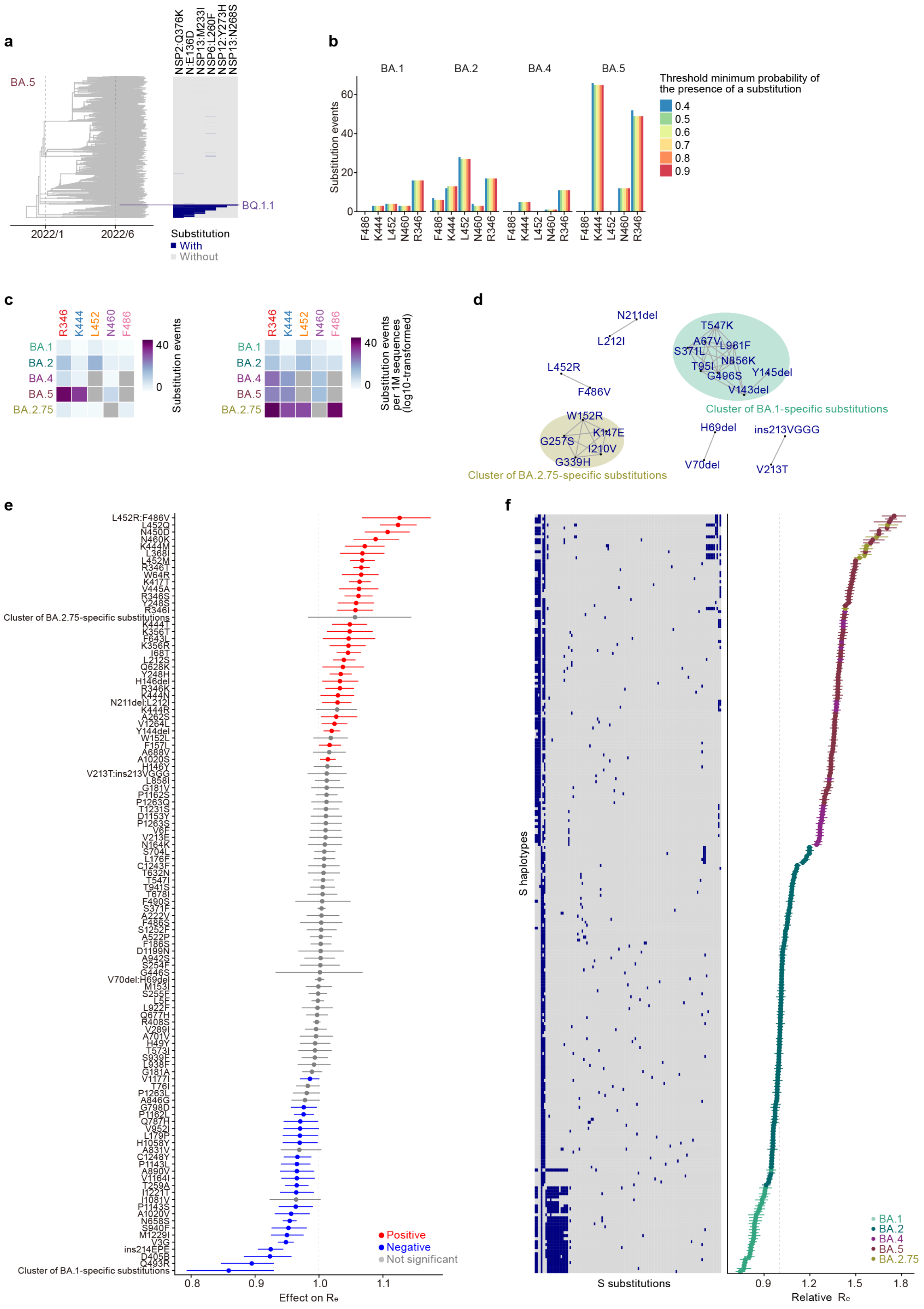

Supplementary Fig. 1. Convergent evolution of the Omicron lineages

a, Non-S substitutions detected in BQ.1.1 compared to the majority of BA.5. Although five non-S substitutions were detected, substitutions other than NSP13:268S were acquired in the ancestral lineages of BQ.1.1 and are not specific to BQ.1.1.

b, A sensitivity analysis on the number of detected substitution events. The minimum probability to define that a substitution exists at each internal node was changed from 0.4 to 0.9 in increments of 0.1, and the number of detected substitution events was counted.
c, Detected substitution events at the convergent sites (related to **Fig. 1d, e**). Raw counts (left) and counts per 1 million (M) analysed sequences (right) are shown. Unlike **Fig. 1d, e**, the results for BA.2.75 are shown in addition to BA.1, BA.2, BA.4, and BA.5.
d, The co-occurrence network of substitutions in the S protein in Omicron lineages. In the S haplotype dataset, a pair of substitutions with Pearson's correlation > 0.9 is considered co-occurring substitutions and indicated as a link in the network. In the modelling analysis, a group of co-occurring substitutions was clustered, and one effect value was estimated for each substitution cluster.
e, Effect size of each substitution in the S protein on relative effective reproduction number (R_e) (related to **Fig. 2b**). A dot and line indicate the posterior mean and the 95% Bayesian confidential interval (CI), respectively.
f, Relative R_e value for a viral group represented by each S haplotype, assuming a fixed generation time of 2.1 days (related to **Fig. 2c**). A dot and line indicate the posterior mean and the 95% Bayesian CI, respectively. Unlike **Fig. 2c**, the profile of all S substitutions analysed is shown on the left side.

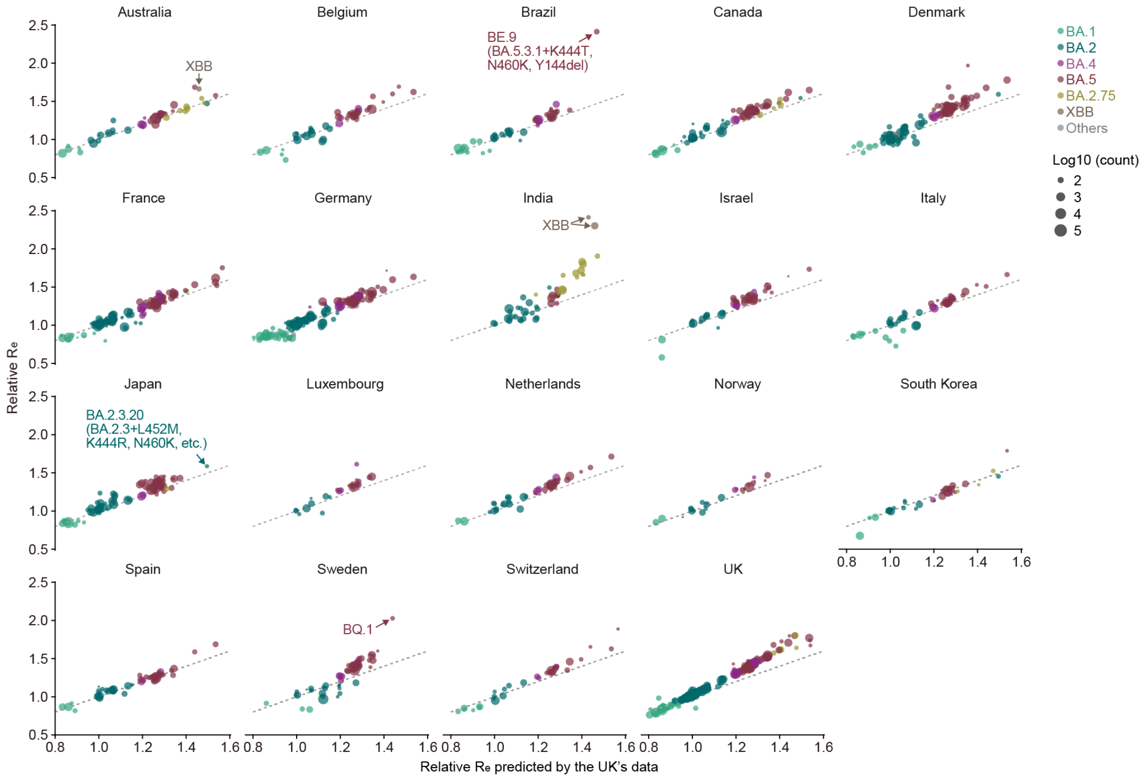

Supplementary Fig. 2. Prediction of the relative R_e of S haplotypes in each country using the model trained on the UK's data

a, The predicted relative effective reproduction number (R_e) of S haplotypes and relative R_e estimated by a multiple logistic model based on each country's data were compared. The dot size indicates the number of sequences of each haplotype. The dotted line denotes a line with a slope of 1 and an intercept of 0. The 20 countries with the highest number of sequences were analysed.

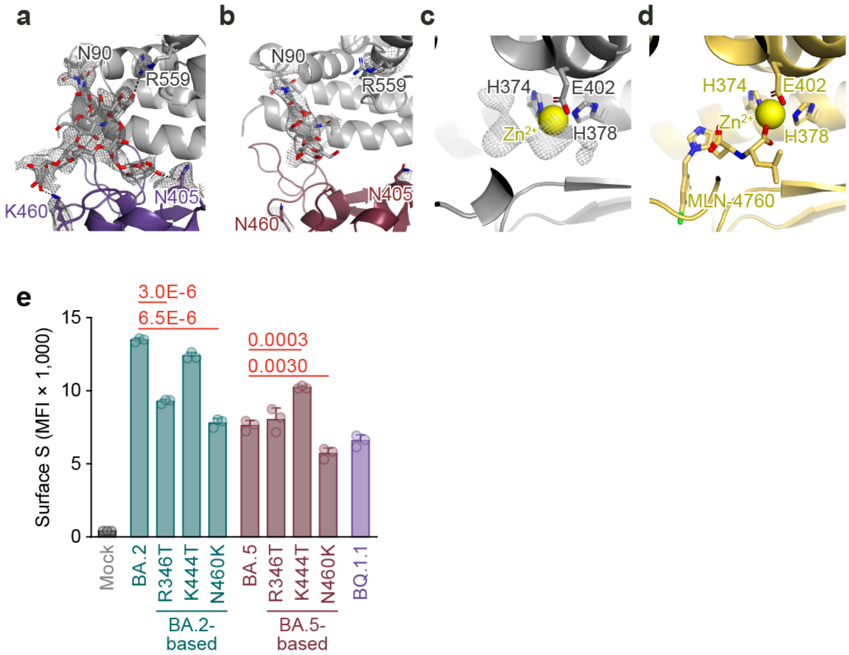

Supplementary Fig. 3. SARS-CoV-2 S–ACE2 interaction

a, b, Electron density maps of the N-linked glycan on Asn90 of human ACE2 bound to BQ.1.1 or BA.4/5 receptor binding domain (RBD). (**a**) BQ.1.1 RBD-human ACE2 complex structure (purple for RBD, grey for ACE2). (**b**) BA.4/5 RBD-human ACE2 complex structure (brown for RBD, grey for ACE2, PDB: 7XWA [https://doi.org/10.2210/pdb7xwa/pdb])[17]. The simulated annealing composite omit map (2*Fo-Fc*) calculated using Phenix is contoured at 1.0σ and shown in grey.

c, d, Structures of the catalytic site of human ACE2. (**c**) The catalytic site of human ACE2 (grey) bound to BQ.1.1 RBD. Electron density (*Fo-Fc* map) of the unidentified substrate-like molecule is shown as grey mesh (contoured at 3.0σ). (**d**) The catalytic site of an inhibitor-bound human ACE2 structure[28] (pale yellow, PDB: 1R4L [https://doi.org/10.2210/pdb1r4l/pdb]).

e, S protein expression on the cell surface. The summarized data are shown. In **e**, assays were performed in triplicate. The presented data are expressed as the average ± standard deviation (SD). Each dot indicates the result of an individual replicate. Statistically significant differences versus each parental S protein were determined by two-sided Student's *t* tests. MFI, median fluorescence intensity.

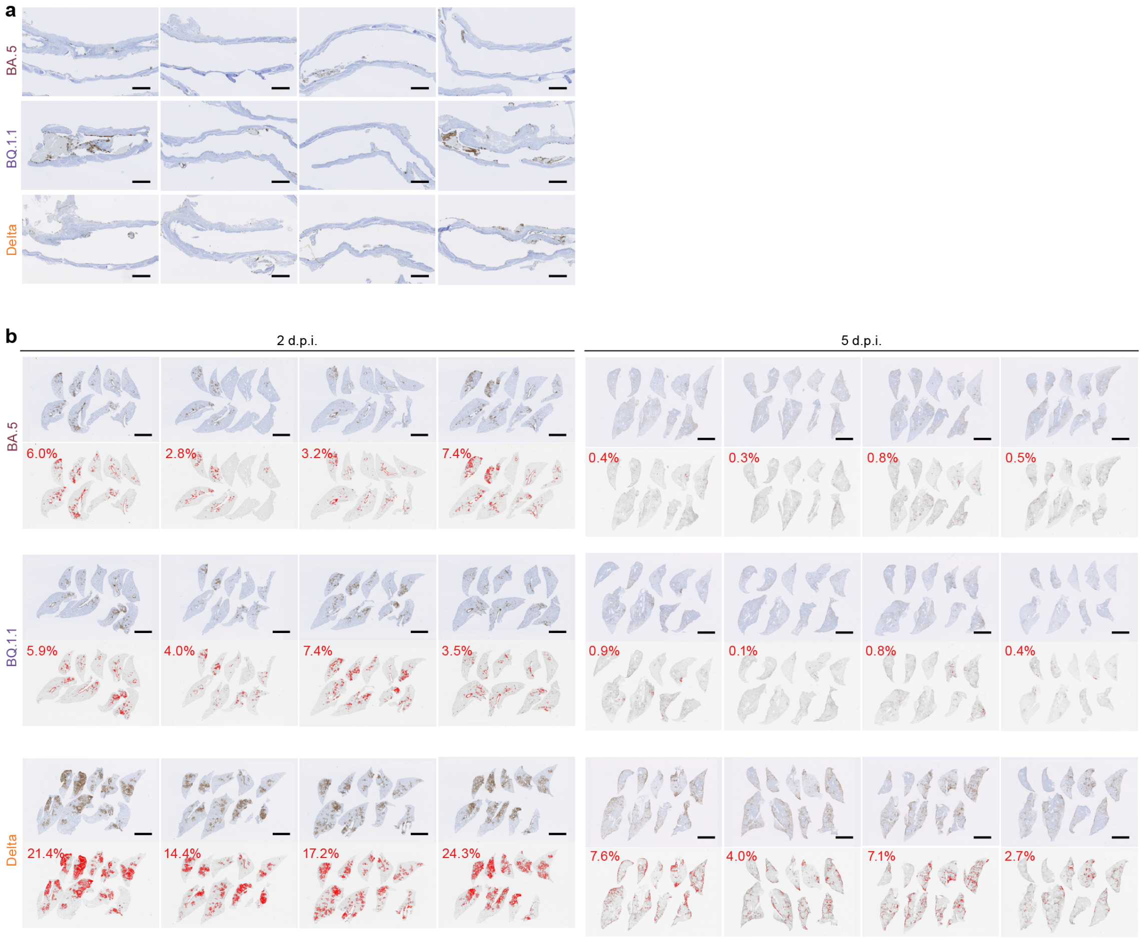

Supplementary Fig. 4. Histological observations in infected hamsters

a, Immunohistochemical (IHC) analysis of the viral N protein in the middle portion of the tracheas of all infected hamsters at 2 days post-infection (d.p.i.) (4 hamsters per infection group). Each panel shows a representative result from an individual infected hamster. d.p.i., days post-infection.

b, IHC analysis of the SARS-CoV-2 N protein in the lungs of infected hamsters at 2 d.p.i. (left) and 5 d.p.i. (right) (4 hamsters per infection group). In each panel, IHC staining (top) and the digitalized N-positive area (bottom, indicated in red) are shown. The red numbers in the bottom panels indicate the percentage of the N-positive area. Summarized data are shown in **Fig. 6c**. In **a and b**, N-positive cells are shown in brown. Scale bars, 1 mm (**a**); 5 mm (**b**).

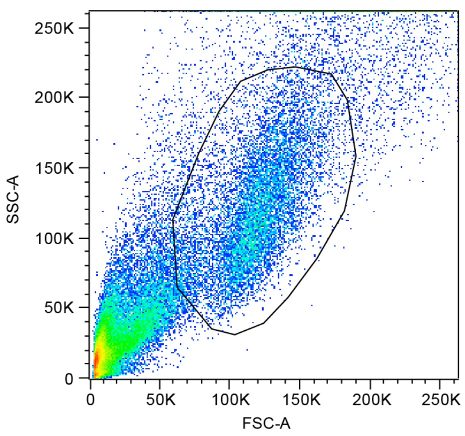

Supplementary Fig. 5. Gating strategy for flow cytometry

A representative dot plot of the gating for flow cytometry is shown. FSC, forward scatter; SSC, side scatter.
