# Supplementary material for: Convergent evolution of SARS-CoV-2 Omicron subvariants leading to the emergence of BQ.1.1 variant
Source: Nat Commun. 2023 May 11;14:2671. doi: 10.1038/s41467-023-38188-z (PMC10175283; doi:10.1038/s41467-023-38188-z)
Supplement: Supplementary file 3 — Description of Additional Supplementary Information [file 41467_2023_38188_MOESM3_ESM.docx]

**Description of additional supplementary files**

Title: Supplementary Data 1

Description: Size of the effect of each substitution in the S protein on R_e_ estimated by a hierarchal Bayesian model

Title: Supplementary Data 2

Description: Relative R_e_ value for a viral group represented by each S haplotype
